# Should cancer treatment be continued during the COVID-19 pandemic? A single Egyptian institution experience

**DOI:** 10.3332/ecancer.2020.1077

**Published:** 2020-07-23

**Authors:** Enas A Elkhouly, Radwa H Salem, Mahmoud Haggag

**Affiliations:** 1 Clinical Oncology Department, Faculty of Medicine, Menoufia University, 23511, Egypt; 2 Microbiology Department, Liver Institute, Menoufia University, 23511, Egypt; 3 General Surgery Department, Faculty of Medicine, Menoufia University, 23511, Egypt

**Keywords:** COVID-19, cancer patients, pandemic, cancer treatment be continued

## Abstract

The first confirmed case of coronavirus disease 2019 (COVID-19) in Egypt was reported on 14 February, 2020. Menoufia Clinical Oncology Centre is at the forefront of delivering care to patients with cancer during this public health crisis in Menoufia Governorate, Egypt. This article highlights the unique circumstances and challenges of cancer treatment during this global pandemic and the importance of organisational structure, preparation and a shared vision for continuing to provide cancer treatment to patients in the face of uncertainty and rapid change.

## Background

The coronavirus disease 2019 (COVID-19), caused by severe acute respiratory syndrome coronavirus 2 (SARS-CoV-2), was first described in Wuhan, China, in December 2019 [1]. On 14 February, 2020 Egypt announced its first COVID-19 case [2]. The virus was announced as a pandemic by the World Health Organization (WHO) on 10 March, 2020. As of 9 May, Egypt announced 8,476 COVID-19 cases and 503 related deaths [3]. Cancer patients have weaker immune systems compared to the general population, both due to the disease itself and the treatment. As such, morbidity and mortality of any serious infections would be expected to be high among cancer patients [4].

The aim of this article is based on the fact that the potential threat of COVID-19 to immunocompromised cancer patients as a result of their disease or the treatment delivered is thought to be significant, so it is of great importance to study the best measures to be used by oncology centres to prevent or limit the exposure of cancer patients to COVID-19 and to provide cancer treatment to patients in need, as safely and as correctly as possible.

There are definitely more questions which needed to be answered during this public health crisis (COVID-19 pandemic) not only those mentioned in the article but also this article will try to discuss and share the experience in pandemic COVID-19 regarding cancer patients in the following points:

- Is there a role for ‘prioritising treatment’ for cancer patients?
- Is there a role for ‘telemedicine’ at the time of the COVID-19 pandemic?
- Can cancer surgeries be delayed or cancelled?
- Can potentially immunocompromising treatments be delayed or altered the delivery?
- The experience of MCOD will be discussed through this article together with a review of the available national and international recommendations regarding this public health crisis (pandemic COVID-19).

After the appearance of COVID-19 in Egypt by mid-February 2020, the staff at Menoufia Clinical Oncology Department have discussed many measures to limit the exposure of cancer patients and medical staff to the coronavirus infection and re-adjustment of the treatment of cancer patients during the COVID-19 pandemic through available national and international regulations although there are limited data at the beginning of March regarding this topic.

## Prioritising treatment for patients with cancer and outpatient considerations during the COVID-19 pandemic

At MCOD, there are four units: A, B and C units for solid tumour oncology and a haemato-oncology unit; each unit has two follow-up (FU) clinics weekly. After the appearance of COVID-19 in Egypt, it should be a must to take a rapid action by MCOD, until the arrival of national strategies from higher authorities, so the outpatients were classified into two groups: Group 1 patients under follow-up either as control or hormonal therapy (HT) and Group 2 patients under active treatments (surgery, chemotherapy (ChT), radiotherapy (RT), biological therapy or immunotherapy) either as neoadjuvant or adjuvant treatment or for metastatic disease. Group 1 under HT had given a medical treatment for the next 4 months by their relatives, and any consultation of them or those under control was done by electronic clinics (E-clinic).

For all patients (Groups 1 and 2), the policy of MCODs focused on health education by providing the following advice:

- Avoid crowded places.
- Wear PPE (personal protective equipment) when you go to the hospital for visits and treatments.
- Wash your hands according to the WHO indications. (There were posters and videos showing this.)
- Do not have contact with friends and relatives with COVID-19 symptoms or those who are living in endemic zones or those coming from abroad from countries with massive pandemic.
- Practice social distancing (at least 1 m) with all people to protect yourself and others.
- Reinforcement of a strict ‘stay at home when ill’ policy and access for testing for symptomatic staff have been applied to limit the exposure of patients.

For Group 1 patients, MCOD’s therapy measures were:

- For patients receiving oral treatment (such as HT), who can be monitored remotely, the drug supply was provided for four courses to reduce hospital visits. Blood monitoring or imaging for those patients can be done in local laboratories and scan centres nearer to home.
- Telemedicine services (such as E-clinics and WhatsApp consultations) were implemented.
- All routine FU visits were delayed.

For Group 2 patients, MCOD’s therapy measures were:

- Patients eligible for surgery have priority access.
- MCOD was ready to accept patients to receive surgery or active chemotherapy/radiotherapy, biological therapy and immunotherapy.
- Outpatient visits for Group 2 patients should be reduced to the safest and most feasible level without compromising their care.
- There should be strict and safe triaging procedures to assess any COVID-19 symptoms and necessity for hospitalisation through lung cancer and haemato-oncology patients.
- Preparation of the checkpoint area at MCOD—through which screening for early detection of potentially infectious cancer patients until they were referred to the main isolation area of the university hospital according to the strategies of the main hospital regulations. Clinical staff responsible for the checkpoint area have been trained and wear PPE.

The abovementioned MCOD strategies are matched with the National Health System defined ‘priorities’ for patients with cancer (Table 1) [5].

## Can cancer surgeries be delayed or cancelled during the COVID-19 pandemic?

- Menoufia University surgical department had cancelled all elective surgeries.
- There are apparent differences in the interpretation of ‘elective surgeries’. At Menoufia surgical department, surgery for invasive malignancies is not elective.

## MCOD’s treatment decision recommendations during the COVID-19 pandemic

- Patients who need radiation should discuss the timing with their radiation oncologist. In some cases, it may be possible to delay treatment without affecting outcomes, but these decisions should be made carefully.
- Shorter radiotherapy courses of radiation may be appropriate when feasible and based on the guidelines.
- Patients who need essential anticancer therapy should still get it, but attempts to de-intensify therapy should continue to avoid the need for hospitalisation.
- Consider the protocols that did not need admission at inpatient floors (such as FOLFOX and FOLFIRI) and be replaced by protocols given at 1-day care unit (such as XELOX and XELIRI) or the use of outpatient infusion pump whenever possible.

## Egyptian Supreme Council of University Hospital recommendations during the COVID-19 pandemic

After about 4 weeks from COVID-19 epidemic in Egypt, the Egyptian Supreme Council of University Hospitals (http://scu.eg/pages/university_hospitals) released the general plan to confront the emerging coronavirus for cancer patients which will be revised every 2 weeks according to the general situation. The main suggestions are as follows:

### Suggestions regarding cancer surgeries during the COVID-19 pandemic

1. Postpone surgery for benign tumours such as goitre, fibroadenoma and parotid adenoma.
2. Postpone surgeries of rehabilitation such as breast reconstruction, free flaps and closure of colostomy.
3. Postpone advanced cases that need major palliative surgery, such as HIPPEC and debulking.
4. Postponing cases with tumour of low malignant potentials.

### Suggestions for solid tumour patients during the COVID-19 pandemic

➢ **General rules:**

- Setting patient levels according to the type of treatment (neoadjuvant, adjuvant and palliative) and the percentage of benefit and risk from treatment and determining the priority level of treatment.
- Determine suitable and affordable alternative treatment methods.
- Determine alternative ways to follow the patient under treatment or the patient who has finished treatment and under follow-up.

➢ **Suggestions:**

1. Choose an oral or subcutaneous treatment instead of intravenous treatment as much as possible.
2. Choose short-term treatment protocols.
3. Choosing the methods of giving chemotherapy every 3–4 weeks, instead of the weekly treatment.
4. Choosing the methods of immunotherapy every 4–6 weeks, instead of every 2–3 weeks.
5. Postponement of palliative treatments (such as Xgeva).
6. Choosing hormonal therapy every 12 weeks instead of every four reasons for prostate tumours.
7. Using GCSF as a preventive treatment with the most common immunodeficiency treatment protocols.
8. Avoid palliative chemotherapy protocols that cause high immunity suppression in the treatment of metastatic tumour cases to avoid the chances of hospitalisation.
9. Discontinue advanced chemotherapy lines (third line or more) in advanced tumour cases with poor treatment results.
10. The patients, who under follow-up after the end of treatment with no new symptoms related to their disease, postpone their follow-up visits for 2 months.
11. Try to dispense hormonal treatment orally enough for the patient for 2–3 months.

### Suggestions regarding the radiation policy during the COVID-19 pandemic

- *Palliation:* hypofractionation policy as follows:
  - Bone metastasis:
    - Single fraction radiotherapy 600–800 cGy/fraction
    - 500 cGy × 3 fractions.
    - 400 cGy × 5 fractions to small radiation field (GTV+ 2 cm margin all around).
    Avoid using multiple sites at the same time, especially in the axial skeleton to avoid bone marrow suppression. Radiate the most painful sites not responding to full-dose analgesics or sites of spinal cord compression or impending fracture.
  - Brain metastasis (if WCI): 400 cGy × 5
  - Lung irradiation (palliative): 300 cGy × 10–13 fractions
- *Breast:* All adjuvant radiotherapy can be delayed except high-risk patients: T3-4, N2-3, TNBC or young age. Adjuvant treatment: 40 Gy/15 fractions + possible concomitant boost if BCS to the site of primary tumour 5 or 8 fractions × 200 cGy.
- *Brain GBM*: hypofractionated schedule 45 Gy/15 fractions concomitant with temozolomide, especially for old-age patients (care of myelosuppression)
- *Prostate:* for radical treatment possible delay up to 6 months if the patient receiving hormonal therapy. Possible hypofractionation IMRT technique, 60 Gy/20 fractions.
- *Rectum:* possible neoadjuvant short-course radiation 500 cGy × 5 fractions to be followed within 1 week by surgery (unless T4b or extension into the anal canal).
- *Lung:* sequential chemoradiotherapy instead of CCRT to delay radiation for 1–2 months and possible use of oral vinorelbine.

### Suggestions for haematology practice during the COVID-19 pandemic

➢ **Rationale:**

1. Minimising the frequency of outpatient clinic (OPC) visits and crowds both in the OPC and day-care units.
2. Minimising the frequency of getting a haematology patient with fever/infection in the outpatient/emergency room (ER) that can be confused with COVID-19 infection.
3. Minimising the frequency of admissions to receive chemotherapy cycles or to treat therapy complications that will increase the hospital inpatient crowd/workload and resources.
4. Give more transplant beds for mandatory transplant patients.
5. Minimise the duration and intensity of immune suppression as more immunosuppressed patients are more likely to have a bad outcome in case they got COVID-19 infection.
6. Haematology staff may be consulted or asked to help in caring COVID-19 patients.

➢ **Suggestions:** shown in Table 2.

### Suggestions for paediatric oncology during the COVID-19 pandemic

1. Patients with ALL due for consolidation to receive two interims (MTX and 6-MP).
2. Patients with ALL on maintenance chemotherapy to receive on month (MTX and 6-MP).
3. Patients with ALL, NHL and relapsed leukaemia to receive TTT on patients and not to be discharged after ending CTH and to receive the next course once recovered.
4. Patients with OS to admitted for P/A and HDMTS.
5. Patients with HR MB to receive cycles with 25% reduction.
6. Patients with H.LCH.E.S, RMS, WT, SR MB and NB will receive treatment at day-care following rules of short, intermediate and long infusion.
7. Palliative cases to receive oral VP16 for 3 weeks.

- All the above suggestions and decisions will be reviewed every 2 weeks, according to the general situation.
- Record the dates of the upcoming visits of all sick patients attending the clinic in a notebook separately, with the renewal of telephone numbers, through the role secretary.
- The number of patients in rooms with five beds will be reduced to two and two patients in rooms with four beds.
- Cases that have completed their surgical, RT and ChT and are preparing for periodic follow-up are postponed for 2 months, and cases are reported by the secretariat, without the patient entering to the doctor, except in cases with bleeding or unexplained high temperature more than 10 days.

## Is there a role for ‘telemedicine’ during the COVID-19 pandemic?

**Figure 1.**
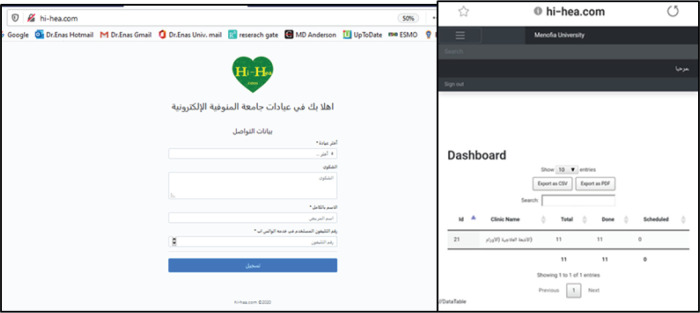
Website of Menoufia University E-clinics and MCOD’s E-clinic visit.

- MCOD had reviewed the schedules of outpatient clinics and identified patients with ‘routine’ follow-up visits whom can be rescheduled. When the patients were contacted to be rescheduled, they are asked whether they have any urgent concerns that need to be addressed before the new appointment. If yes, they were offered a virtual visit.
- Patients who are in long-term follow-up (no evidence of disease at 3 years or longer, being seen annually) or those in routine surveillance after curative treatment (that is, seen every 3 months) are all being contacted, and the visits are being moved to telehealth.
- Any follow-up, non-treatment visits were done by phone calls, WhatsApp consultations or E-clinic which were designed by the Information Technology (IT) unit of Menoufia Faculty of Medicine (MFM). Menoufia University Hospital E-clinics is for all departments including MCOD (http://hi-hea.com). Through the E-clinic link, it is possible through mobile to answer patients’ consultation by oncology staff according to patients’ units (Figure 1).

## MCOD’s Infection Prevention and Control (IPC) Committee instructions during the COVID-19 pandemic

1. Primary care staff, including physicians, nursing and administrative staff with patient contact, should be aware of: a) the current COVID-19 epidemiologic situation in Egypt, globally, b) known risk factors for infections, c) clinical symptoms and signs of COVID-19, d) recommended IPC measures and e) procedures for reporting and transfer of persons under investigation and probable/confirmed cases.
2. Provide signs at all entrances that list the symptoms compatible with COVID-19 (fever, cough and shortness of breath), informing visitors with any of these symptoms not to enter the inpatients’ floor.
3. Ensure that all people within and all who enter the inpatients’ floor practice the appropriate hand hygiene measures, i.e., they should use soap and water or alcohol-based hand rub with considering restricting access to the inpatients’ floor to non-essential visits.
4. Check regularly that all people in the inpatient floor are aware of hand and respiratory hygiene, including cough etiquette with restricting access to the floor.
5. Ensuring that all staff are trained on hand hygiene and standard precautions with signs explaining in all department units.
6. Make alcohol-based hand rub available in all MCOD’s units and all public areas.
7. Ensure that soap dispensers and paper towels are available for handwashing.
8. The number of patients at inpatient rooms with eight beds will be reduced to four and two patients in rooms with four beds (one bed on and one bed off policy).
9. Assess the onsite availability of appropriate personal protective equipment (PPE) for all personnel to apply standard contact and droplet precautions.
10. Consult by telephone or telemedicine/online, if possible, to reduce the number of people with the symptoms of COVID-19, who have contact with health care.
11. A checkpoint room for risk assessment to assess the likelihood of COVID-19 infection, including the clinical presentation of the patient and a review of clinical, epidemiological and travel history. The assessment should be based on the latest case definitions by the Egyptian Ministry of Health.
12. Healthcare workers at MCOD just perform the first assessment without direct contact; in suspected COVID-19 in cancer patients, the patient should wear a surgical mask and keep a distance of at least 1 m before referring the suspected case to the main isolation unit of university hospitals, where the diagnosis is processed.
13. Plan for capacity estimates the needs for patient beds, respiratory support, PPE and alcohol supply.
14. Staff responsible for environmental cleaning and waste management should wear appropriate PPE. If there is an insufficient stock of respirators, then a surgical mask may be worn, as well as gloves and gown. In addition, the use of heavy-duty gloves and boots should be considered.
15. Regular cleaning followed by disinfection is recommended, using hospital disinfectants active against viruses; cleaning inpatient rooms is particularly important for frequently touched surfaces.
16. Regular meeting with main hospital IPC committee for providing additional advice on IPC (such as advice to calculate MCOD’s need for PPE and related products), and discussing the updates of the situation and regulations from the Ministry of Health.

There are definitely more questions that needed to be answered during this public health crisis (pandemic COVID-19) not only those mentioned in the article. Due to the rarity of the situation, this subject needs more and more discussion and sharing for the experience of dealing with this public crisis regarding cancer patients.

## Conclusion

The measures used by MCOD to prevent or limit the exposure of cancer patients to COVID-19 and to provide cancer treatment to patients in need, as safely and as correctly as possible, were as follows: prioritising treatment for cancer patients, telemedicine at the time of the COVID-19 pandemic plays an important role for a selected group of cancer patients, the treatment lines and protocols need to be modulated according to their urgency such as cancer surgeries, potentially immunocompromising treatments, chemotherapy and radiotherapy protocols aim to reduce both the frequency of visits and side effects that need patient admission, and IPC was an essential language for protecting all medical staff and patients.

## Funding

This article was not funded.

## Ethical approval

The study was conducted in accordance with the Declaration of Helsinki and the Ethics Committee of Faculty of Medicine, Menoufia University, approved the study protocol.

## Contributorship

I confirm that all the named authors have participated in the study to a sufficient extent to be named as authors. All authors participate in collecting data and writing according to their specialties and participate in the revision of the paper and approved the final manuscript for submission.

## Declaration of competing interests

The authors declare that they have no conflicts of interest.

## Figures and Tables

**Table 1.** Treatment pathways for patients with cancer in the face of COVID-19.

Setting	Strategy	Measures
Patients’ off treatment	Prevention. Delay visits of follow-up appointments in the absence of active disease. Phone contact or telemedicine consultation.	Community mobilisation. Health education and increased COVID-19 awareness through social media campaigns. Inform patients in the epidemic areas by phone. Suggest protection supplies.
Patients with early-stage cancer in a curative intent (neoadjuvant treatment, surgery and adjuvant treatment)	Prevention. Treat cancer in best of care in hub centres within a ‘COVID-19-free’ clinical pathway.	1–4 listed above. Limit close contact. Cancer team uses personal protective equipment. Close monitoring for potential toxicity and COVID-19 symptoms. ‘COVID-19-free’ clinical pathway.
Patients with metastatic disease.	Prevention. Treat cancer in best of care in ‘hub hospital’ within a ‘COVID-19 free’ clinical pathway.	All measures listed above. Delay treatment if not compromising disease control. If oral therapy is needed, provide drug supply for 2 or 3 courses with hormone monitoring. Use telemedicine for toxicity management.

**Table 2.** Suggestions for haematology practice during the COVID-19 pandemic.

Modifications in non-transplant settings	
DLBCL	Stop R-CHOP at 6 cycles, no benefit from 8 cycles. Consider repeating R-CHOP at 4 weeks if beyond 4 cycles and responding. Consider giving prophylactic G-CSF if prior cycles associated with febrile neutropenia.
HL	Consider giving ABVD at 3 weeks if beyond 4 cycles and responding. Consider giving prophylactic G-CSF if prior cycles associated with febrile neutropenia.
CLL	Patients who finished 4 cycles and responded to FCR or BR can either stop therapy or have the next (5th cycle) postponed by 6 weeks. Responding patients who are on additional maintenance therapy might interrupt/stop therapy for 6–8 weeks.
MM	Consider a change in weekly regimen as VRD/VD/CyBord/CyDex (if oral cyclophosphamide is not available) to oral Len-Dex at a lower dose to allow prescribing 2 cycles with the next visit after 6–8 weeks. Patients on maintenance lenalidomide or bortezomib post-Auto May Interrupt/Stop it for 6–8 weeks. An alternative is to reduce Lenalidomide dose to allow prescribing therapy for 2 months without checking blood counts or a clinic visit.
CML	Consider giving medications for 6–8 weeks if well tolerated in a very stable disease.
PRV	Consider prescribing HU for 8 weeks (some dose reduction can be offered) if very stable disease and good tolerance. 1–2 sessions of Vene-sections can be offered followed by a lowered dose HU can be an option in those with uncertain hematologic tolerance to HU.
ET	Consider giving HU for 8 weeks (some dose reduction can be offered) if very stable disease and good tolerance.
MF	No corresponding explanation for 'MF' was sent from high council.
ALL	In CRs, consider temporary replacing intensive consolidation by more gentle therapy for 4–6 weeks, e.g. 6 weeks of 6MP/MTX maintenance.
AML	In CRs, consider temporary (4–6 weeks) postponing intensive consolidation, e.g. HiDAC or ID ara-C by replacing it with more gentle therapy, e.g. Ara-C 100 mg/m^2^ × 5 days.
AIHA/ITP	Continue the same treatment without trials of dose reductions provided steroid dose is not high. Postpone splenectomy decision for 4–8 weeks if possible.
DLBCL/HL	Auto can be deferred for 2–3 months after the last cycles of effective salvage. Providing an extra cycle of salvage can be an option to allow an additional 2–3 months’ window before proceeding to Auto if this will give a chance for other patients more urgent to Allo.
Benign conditions EXCEPT SAA	Consider delaying Allo for benign haematology conditions, e.g. thalassemia, sickle cell and Fanconi for 6–8 weeks.
MM	Consider cancelling versus a 3-month postponement of front-line Auto in responding myeloma.
ALL	In non-high-risk ALL in CR1 candidate for Allo consider temporary (4-6 weeks) postponing Allo. Giving a bridge maintenance of 6MP/ MTX for 4–8 weeks might be an option.
AML	In non-high-risk AML in CR1 candidate for Allo consider temporary (4-6 weeks) postponing Allo. Giving a bridge cycle of gentle therapy, e.g. Ara-C 120 mg/m^2^ × 5 days might be an option.
